# Effect of peripheral blood absolute monocyte count at admission on relapse-free survival in patients with idiopathic thrombotic thrombocytopenic purpura in remission

**DOI:** 10.3389/fmed.2024.1463086

**Published:** 2024-12-16

**Authors:** Xiaomin Yu, Mingzhu Zhong, Chen Wang, Yifen Shi, Chongyun Xing, Kang Yu, Ying Lin, Rongying Ou, Junjun Yang, Liqing Zhu

**Affiliations:** ^1^Department of Clinical Laboratory, the First Affiliated Hospital of Wenzhou Medical University, Wenzhou, China; ^2^Department of Obstetrics and Gynecology, The First Affiliated Hospital of Wenzhou Medical University, Wenzhou, China; ^3^Department of Hematology, The First Affiliated Hospital of Wenzhou Medical University, Wenzhou, China; ^4^Department of Hematology, The Second Affiliated Hospital of Wenzhou Medical University, Wenzhou, China; ^5^Department of Clinical Laboratory, The Second Affiliated Hospital of Wenzhou Medical University, Wenzhou, China

**Keywords:** Purpura, thrombotic thrombocytopenic, monocyte, prognostic, relapse-free survival

## Abstract

**Introduction:**

Peripheral monocytes have been reported to correlate with clinical outcomes in various types of malignancies. Previous reports have also shown that acute-phase thrombotic thrombocytopenic purpura (TTP) plasma could induce the activation of monocytes. However, the significance of peripheral blood absolute monocyte count (AMC) in idiopathic TTP remains an unanswered question. The primary aim of this retrospective study was to evaluate the prognostic value of AMC at admission in idiopathic TTP patients in remission.

**Methods:**

A total of 37 patients with idiopathic TTP who survived their first episode of the disease and achieved remission following treatment were eligible for inclusion in the study. SPSS and Stata were used to analysis.

**Results:**

There were 1 patient (2.7%) with low AMC (< 0.12  × 10^9^/L), 27 patients (73.0%) with normal AMC (0.12–0.80 × 10^9^/L), and 9 patients (24.3%) with high AMC (> 0.80 × 10^9^/L) at admission. Ten (27.0%) of 37 patients in our cohort subsequently relapsed (1 in the low AMC group and 9 in the normal AMC group). Survival analysis showed that there was a trend of higher relapse-free survival (RFS) rate in patients having increased A MC (log-rank test, *p* = 0.026). Univariate analysis revealed that increased AMC at admission was significantly associated with higher RFS (hazard ratio = 0.12, 95% confidence interval: 0.02–0.62, *p* = 0.011).

**Discussion:**

Our results suggest that increased AMC at admission could represent a predictor of higher RFS in TTP patients having survived their first episode of the disease and achieved remission following treatment.

## 1 Introduction

Thrombotic thrombocytopenic purpura (TTP) is a rare but potentially life-threatening disorder. It is characterized by microvascular thrombosis, microangiopathic hemolytic anemia (MAHA), and consumptive thrombocytopenia (1). The first episode of TTP mostly occurs in adulthood, and approximately 90% of adult cases with TTP are acquired autoimmune disorders (mainly idiopathic) resulting in the production of circulating autoantibodies directed against ADAMTS13, a metalloprotease that cleaves the endothelial cell-derived ultra-large von Willebrand factor (vWF) multimers (2–4). Anti-ADAMTS13 antibodies are mainly immunoglobulin G (IgG), although IgA and IgM have also been reported in some cases (5, 6). Anti-ADAMTS13 antibodies may not only inhibit the enzyme directly (7, 8), but could also cause reduced enzyme activity by enhancing ADAMTS13 clearance from the circulation (9–11). When ADAMTS13 activity is severely reduced (10% or less), platelet hyperadhesive ultra-large vWF multimers accumulate on the endothelial cell surface, favoring the spontaneous formation of platelet-rich microthrombi, which lead to thrombotic microangiopathy (12, 13). The mainstay of treatment for TTP consists of therapeutic plasma exchange, usually in association with glucocorticoid therapy (14). Nevertheless, in TTP patients who achieve remission of the disease, approximately one-third cases are expected to relapse (15). For TTP patients with a high risk of relapse, further immunosuppressive therapy may be considered. For example, a recent study showed that preemptive rituximab could reduce the risk for TTP relapse (16). However, up to now, there are no standard or experimental laboratory tests that can used to reliably predict the risk of TTP relapse.

Monocytes are a key component of both innate and adaptive immunity system. They circulate in the peripheral blood and migrate into tissues, where they can differentiate into tissue macrophages (17–19). Monocytes and their macrophage derivatives play an important role in the regulation of immuno-inflammatory responses (20, 21). Recently, peripheral monocytes have been shown to correlate with clinical outcomes in various types of malignancies (22–26). More relevantly, in autoimmunity diseases such as rheumatoid arthritis, the circulating monocyte counts could be used to predict the clinical treatment response (27). Additionally, previous reports have shown that acute-phase TTP plasma could induce the activation of monocytes (28), which suggest that activated blood monocytes might be implicated in the pathophysiology of TTP. However, the significance of peripheral monocytes in TTP is unknown, and their ability to act as a predictive factor for TTP relapse has not been established. Therefore, the primary purpose of this retrospective study was to evaluate the prognostic value of peripheral blood absolute monocyte count (AMC) in a group of patients with new diagnosed idiopathic TTP, having survived their first episode of the disease and achieved remission following treatment.

## 2 Patients and methods

### 2.1 Patients

The diagnosis of TTP was made based on (i) clinical manifestations characterized by neurological features, renal impairment and fever; (ii) typical laboratory abnormalities including thrombocytopenia, MAHA (anemia, elevated lactate dehydrogenase [LDH], the presence of schistocytes on peripheral smear, and negative Coomb’s test), and normal clotting tests; and (iii) exclusion of Evens syndrome, disseminated intravascular coagulation, HELLP (hemolysis, elevated liver enzymes and low platelets), and eclampsia, hemolytic uremic syndrome (29, 30). The term idiopathic TTP was used to indicate the absence of any of the following identifiable causes: infection, autoimmune disease, exposure to drugs known to cause TTP, surgery, cancer, pregnancy or postpartum period, malignant hypertension, pancreatitis, and stem cell transplantation. Patients with any of the above conditions were considered to have secondary TTP. Eligible patients included adults (16 years and older) with new diagnosed idiopathic TTP, having survived their first episode of the disease and achieved remission following treatment. Patients with congenital TTP or secondary TTP were not included. This retrospective study was approved by the ethics committees of the First Affiliated Hospital of Wenzhou Medical University and the Second Affiliated Hospital of Wenzhou Medical University. The committees waived the patient’s informed consent due to the retrospective nature of this study, but patient confidentiality was protected.

Clinical and laboratory data was retrieved from physical and electronic medical records, including age, gender, initial clinical manifestation, complete blood counts, schistocyte counts on peripheral smears, coagulation tests, serum LDH, serum creatinine, total and indirect bilirubin. The AMC value was determined either by the Sysmex XE-2100 series automated hematology analyzer or manual differential leukocyte counts (in cases the AMC value was flagged as abnormal). In addition, four individual laboratory parameters (platelet count <30 × 10^9^/L, mean corpuscular volume [MCV] < 90 fL, creatinine <176.8 μmol/L, and international normalized ratio [INR] < 1.5), a combined laboratory parameter (evidence for hemolysis based on either reticulocytes >2.5% or indirect bilirubin [IBIL] > 34.2 μmol/L), and two historical features (no active cancer, no history of solid organ or stem-cell transplantation) were collected for calculating the PLASMIC score (31).

Therapeutic plasma exchange and/or plasma infusion was performed in all patients once the diagnosis of TTP was made. Concomitant therapy with glucocorticoids and/or other immunosuppressive drugs was started at the physician’s discretion. Remission was defined as platelet count recovery (> 150 × 10^9^/L) for 2 consecutive days. Relapse was defined as recurrence of an episode of acute TTP beyond 30 days after remission from the first episode of TTP.

### 2.2 Statistical analysis

The chi-squared test was used to compare categorical data, which were expressed as counts and percentages. Continuous data were compared using the Wilcoxon test (U test) and were presented as mean ± standard deviation. Above all were analyzed by SPSS Statistics 22 software (IBM, Armonk, NY). All tests were two-sided. Kaplan–Meier survival analysis was used to plot relapse-free survival curves for patients, and the Log-rank test was applied to compare survival differences between groups. Cox regression analysis was performed to calculate the hazard ratios (HRs) and 95% confidence intervals (95% CIs) to identify risk factors, with *p*-values <0.05 considered statistically significant. These data were analyzed using the Stata version 12 software (StataCorp, College Station, TX).

## 3 Results

### 3.1 Patient characteristics

**Figure 1:**
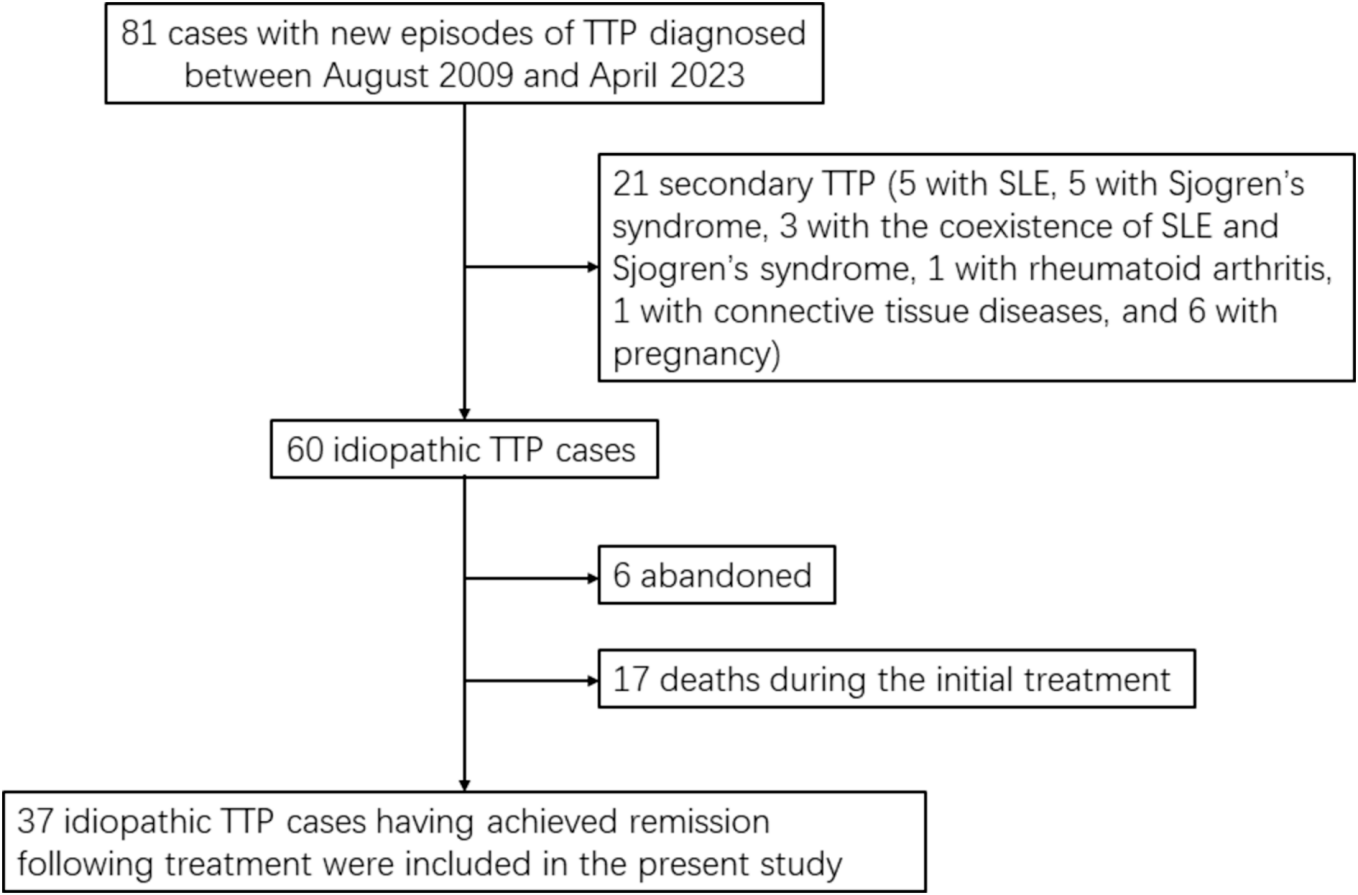
Study flow chart. TTP, thrombotic thrombocytopenic purpura; SLE, systemic lupus erythematosus.

A total of consecutive 81 patients were diagnosed as the first episode of TTP between August 2009 and April 2023 at the First Affiliated Hospital of Wenzhou Medical University and the Second Affiliated Hospital of Wenzhou Medical University. There was no family history of the disease in any of the 81 TTP patients. 21 patients presented with concomitant conditions associated with secondary TTP (five patients with systemic lupus erythematosus [SLE], five with Sjogren’s syndrome, three with the coexistence of SLE and Sjogren’s syndrome, 1 with rheumatoid arthritis, 1 with connective tissue diseases, and six with pregnancy), six refused plasma exchange or plasma infusion after diagnosis, and 17 died from disease progression during the initial treatment with plasma exchange. Thus 37 patients with idiopathic TTP who survived their first episode of the disease and achieved remission following treatment were eligible for the evaluation of risk factors for relapse (Figure 1).

The characteristics of the 37 idiopathic TTP patients are shown in Table 1. The patient cohort consisted predominantly of females (59.5%), with a median age of 51 years. Neurologic symptoms occurred in 78.4% of the patients, and 56.8% had fever. Of the 37 patients with idiopathic TTP, the median hemoglobin concentration and platelet count were 75 g/L and 19 × 10^9^/L, respectively. The schistocyte percentage was obviously increased with a median of 8.3%. The median serum LDH and indirect bilirubin level at admission were 854 IU/L and 25.2 μmol/L. 35 patients had necessary data for calculation of the PLASMIC score. A total of 22 (62.9%) and 13 (37.1%) patients had a PLASMIC score of 5 and 6 or 7, respectively. The time elapsed from the onset of initial symptoms to initiation of therapy ranged from 1 to 64 days, with a median of 13 days. In this series, all patients received plasma exchange, with the exception of one patient who was treated with plasma infusion and the other seven patients who were initially treated with plasma exchange and subsequently treated with plasma infusion. All patients also received glucocorticoids as adjunctive treatment. Other therapies used to achieve remission were rituximab (*n* = 4), mycophenolate mofetil (*n* = 17), cyclosporine A (*n* = 6), and cyclophosphamide (*n* = 1) in 25 patients. Within our entire cohort, the median time from initiation of therapy to remission was 11 days.

**Table 1:** Clinical and treatment data for the patient cohort [median (range) or n (%)].

	All patients (*n* = 37)	Low AMC (< 0.12 × 10^9^/L) (*n* = 1)	Normal AMC (0.12–0.80 × 10^9^/L) (*n* = 27)	High AMC (> 0.80 × 10^9^/L) (*n* = 9)	*p* value^£^
Age (years)	51 (16–79)	69	49 (16–78)	56 (30–79)	0.289
Female	22 (59.5)	1	14 (51.9)	7 (77.8)	0.172
Clinical manifestations					
Fever	21 (56.8)	0	15 (40.5)	6 (66.7)	0.558
Neurologic symptoms	29 (78.4)	0	22 (81.5)	7 (77.8)	0.808
Laboratory values					
Hemoglobin (g/L)	75 (34–107)	78	75 (34–107)	74 (50–95)	0.855
Platelets (×10^9^/L)	19 (3–64)	7	20 (6–64)	17 (3–38)	0.884
WBC count (×10^9^/L)	9.51 (2.69–29.40)	9.27	8.20 (2.69–24.52)	13.47 (5.94–29.40)	0.011
Schistocyte^*^ (%)	8.3 (0.8–31.2)	15.2	8.2 (1.2–31.2)	7.7 (0.8–16.0)	0.761
D-dimer^†^ (mg/L)	2.02 (0.35–6.64)	0.63	2.12 (0.35–6.64)	1.91 (1.07–4.08)	0.543
LDH^‡^ (IU/L)	854 (236–1888)	704	845 (236–1,688)	895 (453–1888)	0.891
Creatinine (μmol/L)	93.1 (35.0–307.0)	110	83.3 (35.0–289.0)	120.8 (58.0–307.0)	0.108
Total bilirubin (μmol/L)	35.4 (11.0–108.7)	62	35.3 (11.0–108.7)	32.4 (18.0–55.0)	0.855
Indirect bilirubin (μmol/L)	25.2 (7.0–101.4)	42	25.6 (7.0–101.4)	22.0 (11.0–30.0)	0.714
PLASMIC score^#^					
≤5	22 (62.9)	0	14 (56.0)	8 (88.9)	0.077
6 or 7	13 (37.1)	1	11 (44.0)	1 (11.1)	
Initial treatment					
Plasma exchange	29 (78.4)	1	20 (74.1)	8 (88.9)	0.355
Total plasma exchange volume^§^ (ml/kg)	176.30 (19.85–616.67)	428.40	151.44 (19.85–320.00)	225.42 (64.00–616.67)	0.212
Plasma infusion	1 (2.7)	0	1 (3.7)	0	0.537
Plasma exchange+ plasma infusion	7 (18.9)	0	6 (22.2)	1 (11.1)	0.399
Glucocorticoids	37	1	27	9	–
Second-line treatment					
Rituximab	4 (10.8)	0	3 (11.1)	1 (11.1)	–
Mycophenolate mofetil	17 (45.9)	1	12 (44.4)	4 (44.4)	–
Cyclosporine A	6 (16.2)	0	5 (18.5)	1 (11.1)	0.606
Cyclophosphamide	1 (2.7)	0	1 (3.7)	0	0.537
Time from onset of symptoms to admission (days)	12 (1–63)	30	9 (1–60)	16 (4–63)	0.186
Time from admission to initiation of therapy (days)	0 (0–4)	2	0 (0–4)	0 (0–3)	0.484
Time from onset of symptoms to initiation of therapy (days)	13 (1–64)	32	10 (1–60)	17 (4–64)	0.175
Time from initiation of therapy to remission (days)	11 (2–40)	20	12 (3–40)	8 (2–21)	0.350

£ Comparison between normal and high AMC groups.

† value available for 27 patients.

‡ value available for 35 patients.

# value available for 35 patients.

§ value available for 29 patients.

*value available for 28 patients. AMC, absolute monocyte count; WBC, white blood cell; LDH, lactate dehydrogenase.

According to the lower (0.12 × 10^9^/L) and upper limits (0.80 × 10^9^/L) of normal AMC value, there were 1 patient (2.7%) with low AMC, 27 patients (73.0%) with normal AMC, and 9 patients (24.3%) with high AMC at admission. Due to only 1 patient with low AMC in our cohort, we further compared the patient characteristics between patients with normal and high AMC at admission (Table 1). Patients with high AMC tended to associate with high WBC count (*p* = 0.011).

### 3.2 Prognostic impact of AMC at admission

Ten (27.0%) of 37 patients in our cohort subsequently relapsed (1 in the low AMC group and 9 in the normal AMC group), with a median time to relapse of 17.5 months (range: 2.7–87.0 months) from remission. Most relapses (7 patients, 70.0%) occurred within the first years after remission. With a median follow-up duration of 44.3 months (range: 0.7–124.7 months) from the data of remission to the date of the last follow-up visit, the estimated 5-year rate of RFS for the entire cohort was 72.60% (95% confidence interval [95% CI], 47.62–87.09%). No patients died during the follow-up.

**Figure 2:**
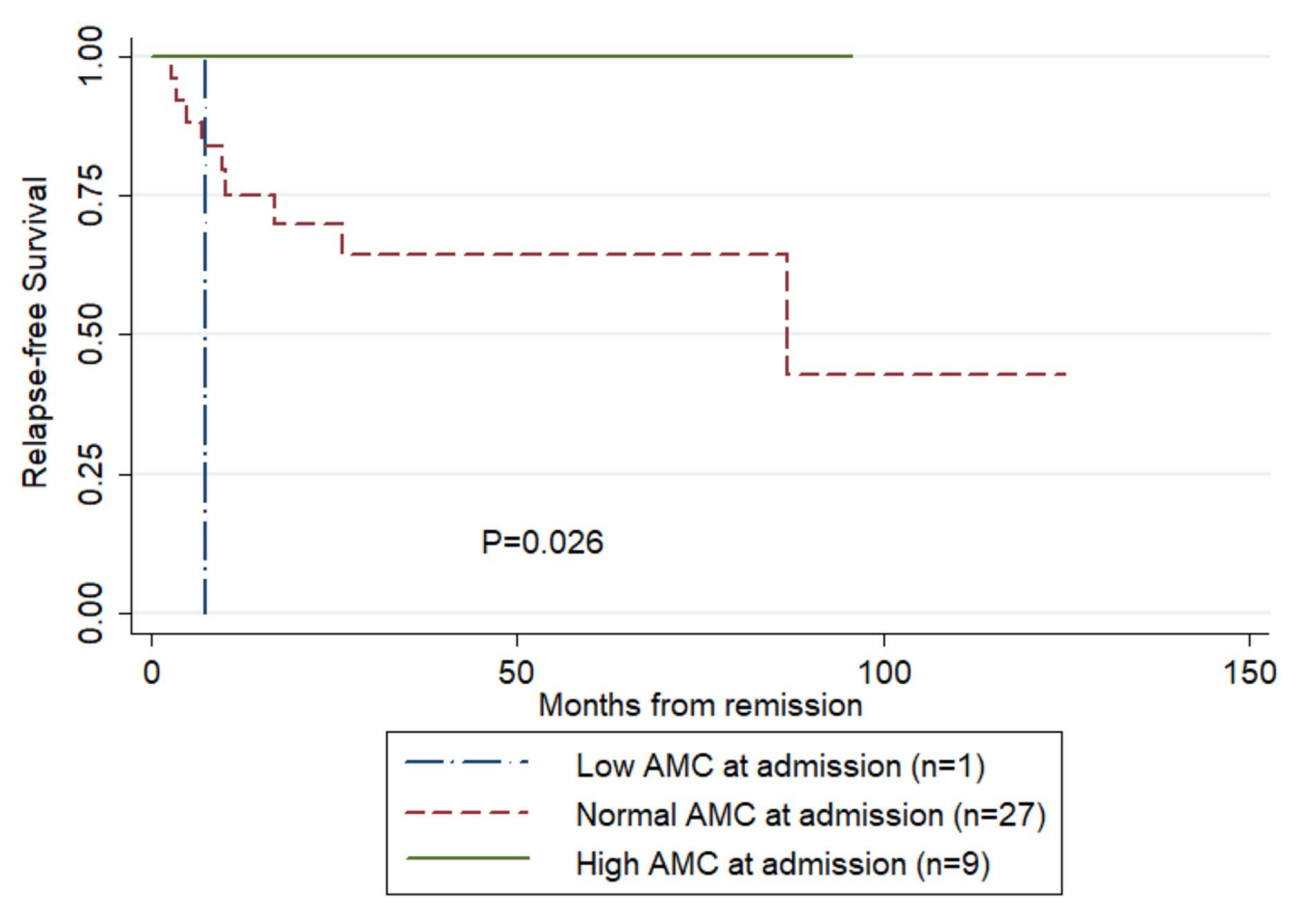
Comparison of relapse-free survival rates in the patients having survived their first episode of TTP and achieved remission following treatment (Kaplan–Meier curves). AMC, absolute monocyte count.

Figure 2 compares RFS curves among the three AMC groups of patients. There was a trend of higher RFS rate in patients having increased AMC (log-rank test, *p* = 0.026). Results of the Cox univariate analysis for risk factors for RFS are reported in Table 2. When AMC was transformed to ordinal variable and ordered as 1 (low AMC), 2 (normal AMC) and 3 (high AMC), increased AMC at admission was significantly associated with higher RFS (hazard ratio [HR] = 0.12, 95% CI: 0.02–0.62, *p* = 0.011). None of the other parameters analyzed were significant (*p* > 0.05), so multivariate analysis was not performed.

**Table 2:** Univariate analysis for relapse-free survival in patients with TTP in remission.

Variate	*p* value	HR (95% CI)
Increased AMC^*^	0.011	0.12 (0.02–0.62)
Age (years)	0.361	1.00 (0.97–1.05)
Female	0.671	0.79 (0.20–3.16)
Fever	0.222	1.91 (0.48–7.68)
Neurologic symptoms	0.306	0.39 (0.09–1.63)
Hemoglobin (g/L)	0.824	1.00 (0.96–1.04)
Platelets (×10^9^/L)	0.404	0.96 (0.90–1.04)
WBC count (×10^9^/L)	0.940	1.01 (0.89–1.16)
Schistocyte (%)	0.539	1.03 (0.94–1.13)
D-dimer (mg/L)	0.073	0.18 (0.03–1.24)
LDH (IU/L)	0.575	1.00 (0.99–1.00)
Creatinine (μmol/L)	0.251	0.98 (0.95–1.00)
Total bilirubin (μmol/L)	0.474	0.99 (0.94–1.03)
Indirect bilirubin (μmol/L)	0.274	0.98 (0.92–1.05)
PLASMIC score (5 versus 6 or 7)	0.404	1.28 (0.37–4.45)
Total plasma exchange volume (ml/kg)	0.298	1.00 (0.99–1.00)
Second-line treatment	0.664	2.33 (0.48–11.4)
Time from onset of symptoms to initiation of therapy (days)	0.298	1.01 (0.96–1.07)
Time from initiation of therapy to remission (days)	0.549	1.02 (0.96–1.09)

*AMC was transformed to ordinal variable and ordered as 1: Low AMC; 2: Normal AMC; 3: High AMC. AMC, absolute monocyte count; HR, hazard ratio; 95% CI, 95% confidence interval; WBC, white blood cell; LDH, lactate dehydrogenase.

## 4 Discussion

As far as we know, this is the first study investigating the prognostic value of AMC at admission in a cohort of 37 newly diagnosed idiopathic TTP, having survived their first episode of the disease and achieved remission following treatment. Our study demonstrated that increased AMC at admission represented a predictor of higher RFS rate in TTP. The association between AMC at admission and RFS in TTP makes it possible that the AMC, the commonly used laboratory parameter, may be conveniently incorporated into a prognostic index for TTP.

The intrinsic correlation between AMC and RFS in TTP patients might attribute to myeloid derived suppressor cells (MDSCs), negative regulators of immune responses. It is well known that chronic inflammation states, including autoimmune diseases, infections, and cancer, could induce the expansion and accumulation of MDSCs (32). In addition, previous studies have shown that peripheral blood monocytes or mononuclear cells cocultured with tumor cells may acquire immunosuppressive, MDSC-like properties (33–35). More relevantly, Alvarez-Larran et al. found that plasma from the patients in acute phase of TTP could induce reactive oxygen species (ROS) production by monocytes (28). Since the authors did not conduct phenotypic and functional analysis within the monocyte-derived cell population, it is not clear if the monocytes also acquired MDSC-like properties. However, it is possible for a positive correlation between peripheral monocytes (%) and MDSCs, as shown in diffuse large B-cell lymphoma (36). Additionally, release of ROS is one of the mechanisms that MDSCs used to negatively regulate B cell responses (37, 38). What’s more, other effector molecules expressed or secreted by MDSCs, such as PD-1/PD-L1, interleukin 10, tumor growth factor-*β*, prostaglandin E2, arginase-1, indoleamine-pyrrole 2,3-dioxygenase, nitric oxide, and cysteine, also involve inhibition of B cell responses (37, 39–41). Furthermore, MDSCs have been shown to act directly on B cells to reduce antibody production through V-domain Ig suppressor of T-cell activation (42, 43), or by cell-to-cell interaction (44). Since the presence of anti-ADAMTS13 inhibitor at presentation has been reported to be associated with increased likelihood of TTP relapse (45, 46), then it is not surprising that increased AMC at admission might reflect the expansion of MDSCs in TTP patients during acute episodes, which in turn associates with suppression of B cell responses, a decrease of anti-ADAMTS13 antibody production, an increase in ADAMTS13 activity and an improvement in RFS. Nevertheless, in order to examine the above possible immunologic mechanisms underlying the prognostic value of AMC in TTP, future quantitative and functional studies are needed to investigate the association of the AMC at admission with MDSCs density, as well as ADAMTS13 inhibitor level and ADAMTS 13 activity.

Our study has some limitations. As a retrospective study with small sample, potential selection bias should be taken into account. Therefore, further well-designed randomized clinical trials or observational studies of large patient populations are required to confirm our results. In addition, of the 60 idiopathic TTP patients receiving therapeutic plasma exchange and/or plasma infusion, 17 (28.3%) patients died from TTP progression during the initial treatment; the mortality of which was much higher in comparison to the 10–20% generally published with appropriate therapeutic management over the last 2 decades (14, 30). Possible contributors to the high mortality include delay in admission and insufficient plasma exchange and/or plasma infusion performed.

## 5 Conclusion

This study for the first time correlates the AMC at admission with RFS in patients having survived their first TTP episode and achieved remission following treatment. Additionally, we report the AMC at admission predictive of relapse that, if validated in other patient populations, may help identify TTP patients in remission who need for close follow-up or in whom more aggressive immunosuppressive therapy should be considered.

## Data Availability

The data analyzed in this study is subject to the following licenses/restrictions: The datasets generated for this study are available on request to the corresponding author. Requests to access these datasets should be directed to Liqing Zhu, zhuliqing@wzhospital.cn.
